# Effect of initial temperature changes on myocardial enzyme levels and cardiac function in acute myocardial infarction

**DOI:** 10.3892/etm.2014.1678

**Published:** 2014-04-14

**Authors:** YUANYU QIAN, JIE LIU, JINLING MA, QINGYI MENG, CHAOYING PENG

**Affiliations:** Department of Emergency, Chinese PLA General Hospital, Beijing 100853, P.R. China

**Keywords:** acute myocardial infarction, body temperature, myocardial enzyme, cardiac function

## Abstract

In the present study, the effect of initial body temperature changes on myocardial enzyme levels and cardiac function in acute myocardial infarction (AMI) patients was investigated. A total of 315 AMI patients were enrolled and the mean temperature was calculated based on their body temperature within 24 h of admission to hospital. The patients were divided into four groups according to their normal body temperature: Group A, <36.5°C; group B, ≥36.5°C and <37.0°C; group C, ≥37.0°C and <37.5°C and group D, ≥37.5°C. The levels of percutaneous coronary intervention, myocardial enzymes and troponin T (TNT), as well as cardiac ultrasound images, were analyzed. Statistically significant differences in the quantity of creatine kinase at 12 and 24 h following admission were identified between group A and groups C and D (P<0.01). A significant difference in TNT at 12 h following admission was observed between groups A and D (P<0.05), however, this difference was not observed with groups B and C. The difference in TNT between the groups at 24 h following admission was not statistically significant (P>0.05). Significant differences in lactate dehydrogenase at 12 and 24 h following admission were observed between groups A and D (P<0.05), however, differences were not observed with groups B and C (P>0.05). Significant differences in glutamic-oxaloacetic transaminase at 12 and 24 h following admission were observed between groups A and D (P<0.05), however, differences were not observed in groups B and C (P>0.05). However, no significant differences were identified in cardiac function index between all the groups. Therefore, the results of the present study indicated that AMI patients with low initial body temperatures exhibited decreased levels of myocardial enzymes and TNT. Thus, the observation of an initially low body temperature may be used as a protective factor for AMI and may improve the existing clinical program.

## Introduction

Coronary heart disease is currently a serious threat to human health. The extent of myocardial infarction (MI) is important when assessing the therapeutic strategy and evaluating the prognosis for acute MI (AMI) patients (1–3). Positron emission tomography (PET) is regarded as the gold standard for identifying viable myocardia. However, there are disadvantages associated with PET, including the requirement for expensive specialist equipment and the complexity of continuous detection and popularization. Thus, a simple and inexpensive method for the continuous detection of the degree of MI is required. As MI is an inflammatory process and body temperature is an inflammatory indicator that is easily obtained in clinical practice, the initial change in body temperature in AMI patients may be a potential indication of the degree of MI (4–6). An animal study conducted using mice demonstrated that genetic background, gender, age (but not in imprinting control regions mice), body temperature and arterial blood pH exhibit considerable effects on infarction size (7). However, clinical studies have only determined the ambient temperature as a risk factor. An additional study demonstrated that no association existed between the daily ambient temperature and the prognosis of AMI, with and without adjusting for PM_10_, NO_x_, O_3_, age and gender (8). Furthermore, an additional study demonstrated that exposure to cold temperatures increased the risk of acute MI (9) and acute aortic dissection (10), whereas heat exposure increased the risk of mortality following AMI (9).

The present study was conducted with hospitalized AMI patients to investigate the changes in myocardial enzyme levels and cardiac function in association with differing body temperature gradients.

## Patients and methods

### Patients

A total of 385 AMI patients were admitted to the Chinese PLA General Hospital (Beijing, China) between 2001 and 2010. The patient group comprised 315 males (81.8%) and 70 females (18.2%). The age of the subjects ranged between 27 and 68 years with a mean age of 49.25±7.45 years. Body temperature was measured within 24 h of admission at intervals of 4 h and the mean temperature was calculated. Subjects were divided into four groups based on their normal body temperature: Group A, <36.5°C; group B, ≥36.5°C and <37.0°C; group C, ≥37.0°C and <37.5°C and group D, ≥37.5°C.

The sample sizes of groups A–D were 83 (males, 62; females, 21), 153 (males, 133; females, 20), 963 (males, 72; females, 24) and 53 (males, 48; females, 5), respectively. The age ranges of groups A–D were 28–65, 27–68, 27–67 and 27–68 years, respectively, with the mean age of groups A–D being 51.21±3.4, 49.86±3.1, 50.48±3.3 and 50.44±3.1 years, respectively. The present study was conducted in accordance with the Declaration of Helsinki and with approval from the Ethics Committee of the Chinese PLA General Hospital. Written informed consent was obtained from all participants.

### Diagnostic criteria

Inclusion criteria were determined based on the diagnostic criteria of MI, including: i) Chest pain that persisted for >30 min; ii) an electrocardiogram that exhibited elevation of the last two connected precordial ST-segments; iii) serum enzyme levels indicating that the creatine kinase-MB fraction (CK-MB) was significantly higher than normal levels; and iv) the patient had no history of MI. The exclusion criteria were: i) Onset of AMI >24 h prior to admission and ii) the patient exhibited a co-infection, malignant disease or severe liver or kidney failure. The blood flow index, vascular recanalization index and the degree of symptom relief was based on the Seventh American College of Chest Physicians Conference on antithrombotic and thrombolytic therapy. A total of 315 patients were included in the present study.

### Data collection

The body temperature of each patient was measured within 24 h of admission to hospital at intervals of 4 h. The mean axillary temperature was calculated and the patients were assigned to different groups based on their normal body temperature. Information was collected from each patient, including age, gender and risk factors of coronary heart disease (smoking, hypertension, diabetes and hypercholesterolemia). At 12 and 24 h following admission, the blood samples obtained from the patients were tested for CK, CK-MB, cardiac troponin T (TNT), lactate dehydrogenase (LDH) isoenzyme and glutamic-oxaloacetic transaminase (GOT). The myocardial enzyme levels were tested using a Roche test kit (Roche Ltd.; Shanghai, China). At 24 h and one week following admission, the patients were examined using Vivid 7 Dimension color Doppler flow imaging (General Electric Company, Schenectady, NY, USA) and the cardiac function indexes were observed.

### Statistical analysis

The mean and SD were calculated to analyze the measured data and comparisons between the groups were tested using analysis of variance and χ^2^ tests, whereas the count data were analyzed with ratios. Pearson’s correlation analysis was used to analyze the correlations between body temperature and the corresponding index. The data were cleaned and analyzed using SPSS 11.0 (SPSS, Inc., Chicago, IL, USA). P<0.05 was considered to indicate a statistically significant difference.

## Results

### Distribution of MI areas

Tables I and II show the general distribution of the MI areas that were observed in each group. In total, 315 patients received interventional examinations within 4 h of admission to hospital. A total of 144 cases of single coronary artery lesion, 116 cases of double branch and 125 cases of triple branch artery lesions were reported. The predominant areas of MI were the anterior descending artery and the right coronary artery; the anterior descending artery lesion was mainly observed in the proximal and middle branches. Thus, the anterior descending artery lesion was the main type of MI, followed by the right coronary artery and the circumflex artery. The patients received stent implantations and the distribution of the MI areas in the four groups was estimated using a χ^2^ test. The comparability for the other variables was reasonable as no significant differences were observed between the groups.

### Body temperature changes

Body temperature was measured within 24 h of admission to hospital at intervals of 4 h. Among the four groups, the majority of patients were in groups B and C, thus, body temperature predominantly ranged between 35.5 and 37.0°C (Table III).

### Myocardial enzyme changes

Myocardial enzyme levels in each group were measured and compared at 12 and 24 h following admission to hospital. With regard to the CK level at 12 h following admission, significant differences were identified between group A and groups C and D (P<0.01), as well as between group B and groups C and D (P<0.05). However, no significant differences were observed between groups A and B (P>0.05) or between groups C and D (P>0.05). With regard to the CK level at 24 h following admission, significant differences were observed between group A and groups C and D (P<0.01), as well as between group B and groups C and D (P<0.05). No significant differences were identified between groups A and B (P>0.05) or between groups C and D (P>0.05; Table IV).

With regard to the CK-MB level at 12 h following admission, significant differences were observed between group A and groups C and D (P<0.01), as well as between group B and groups C and D (P<0.05). However, no significant differences were identified between groups A and B (P>0.05) or between groups C and D (P>0.05). With regard to the CK-MB level at 24 h following admission, significant differences were observed between group A and groups C and D (P<0.01), as well as between group B and groups C and D (P<0.05). However, no significant differences were observed between groups A and B (P>0.05) or between groups C and D (P>0.05; Table IV).

The difference in TNT at 12 h following admission was significant between groups A and D (P<0.05), however, was not significant between group A and groups B and C. The difference in TNT at 24 h following admission among the four groups was not identified to be statistically significant (P>0.05; Table IV).

Significant differences in LDH at 12 h following admission were observed between groups A and D (P<0.05), however, were not observed between group A and groups B and C (P>0.05). Significant differences in LDH at 24 h following admission were observed between groups A and D (P<0.05), but not between group A and groups B and C (P>0.05; Table IV).

Significant differences in GOT at 12 h following admission were observed between groups A and D (P<0.05), but not among other groups (P>0.05). Significant differences in GOT at 24 h following admission were observed between groups A and D (P<0.05), but similarly were not observed among the other groups (P>0.05; Table IV).

### Changes in cardiac function

All 315 AMI patients were examined via cardiac ultrasound at 24 h and one week following admission. Table V shows the comparisons between the main cardiac function indexes. No statistically significant differences were identified between the groups, indicating that initial body temperature changes in AMI did not effect cardiac function.

## Discussion

The initial phase of AMI is the ischemic state, where ischemic myocardial cells maintain vitality, but gradually die over time. A greater quantity of ischemic myocardial cells survive when acute myocardial ischemia reperfusion therapy is conducted at an earlier stage, regardless of intravenous thrombolysis or interventional therapy. However, few AMI patients are able to receive thrombolytic and interventional therapy in a timely manner and the majority of patients receive reperfusion therapy after 3 h of chest pain (11). Therefore, protection from myocardial ischemia and delaying myocardial necrosis are essential. MI is an inflammatory process and body temperature is an inflammatory indicator that is easily acquired in clinical practice. The initial changes in body temperature in AMI patients may be positively associated with the degree of MI (2). The correlation between initial body temperature in AMI and the MI area has been increasingly investigated in previous years (12,13); however, the present study analyzed the effect of initial temperature on AMI in association with myocardial enzyme reactions.

Previous clinical studies have identified differences in the expression of myocardial enzymes in patients at different body temperatures. CK level in AMI patients increases between 4 and 8 h after MI and subsequently decreases over 4–5 days (14). In the present study, the CK and CK-MB levels in patients were significantly lower in group A than those observed in group D. In addition, the other indexes were lower in groups A–C when compared with those in group D. Thus, an increase in body temperature was shown to be associated with mean levels of myocardial enzymes. Patients with lower body temperatures exhibited lower levels of myocardial enzymes and had an improved clinical prognosis, whereas patients with higher body temperatures received a poor prognosis. Elevated body temperature may be a manifestation of the inflammatory reaction following myocardial necrosis. The inflammatory reaction has an adverse effect on left ventricular remodeling (15). However, in a study of 171 AMI patients, in which only 17 patients exhibited a peak body temperature of >37.5°C, no significant correlation was identified between the peak body temperature and the predetermined inflammatory response markers (16). Although an elevated temperature is a natural reaction in the process of repair, it may be associated with increased activation of the immune system, which promotes left ventricular remodeling (17). Body temperature has a marked effect on oxygen consumption. Oxygen demand increases with increasing body temperature, thus, a fever may affect the infarction area. An association between elevated body temperature and elevated CK levels was determined in the present study. A study of 357 AMI patients showed that the anterior wall of the MI area significantly narrowed in AMI patients with a lower body temperature, which was accompanied by low CK-MB levels and an increased left ventricular ejection fraction. By contrast, the treatment outcomes were unaffected by low body temperatures observed in the control group (18,19).

Previous studies have identified that numerous factors affect the prognosis of AMI patients. Body temperature monitoring is a simple method with the ability to estimate risks and the prognosis of AMI patients. The effect of low body temperature on AMI is currently being investigated via animal experimentation (20). Data indicate that hypothermia exhibits a protective effect on the myocardial ischemia model. A previous study investigated whether the use of warm or cold blood cardioplegia resulted in superior myocardial protection. Reduced cardiac enzyme release and improved postoperative cardiac indexes were observed in the warm cardioplegia group, however, similar short-term mortality was identified with no differences observed in the clinical outcomes (21). The application of this measure in clinical practice is currently difficult predominantly due to the lack of effective safety methods to cool the heart (22–24). In addition, clinical studies have identified that numerous factors affect body temperature. Thus, full consideration of the confounding factors is required and cases that include bias factors should be excluded.

In conclusion, few studies have investigated the effect of body temperature in AMI patients on MI recovery in China. To the best of our knowledge, this is the first study to investigate whether hypothermia therapy reduces the MI area, inflammatory reaction and immune response; therefore, these areas may be the subject of future investigations.

## Figures and Tables

**Table I:** Distribution of the myocardial infarction area.

Area	Cases	Proximal	Middle	Distal	Proximal-middle	Mid-distal	Proximal-mid-distal
Anterior descending	315	125	110	9	41	21	10
Circumflex	196	42	77	31	15	21	10
Right coronary	242	53	63	55	20	36	15

**Table II:** Distribution of the myocardial infarction area observed in each group.

Group	Anterior descending	Circumflex	Right coronary
A	65	43	51
B	121	56	86
C	87	67	73
D	42	30	32

P>0.05; χ^2^=0.215.

**Table III:** Distribution of patients in each group.

	Mean body temperature within 24 h of admission to hospital, °C			
	
	<36.5	≥36.5, <37.0	≥37.0, <37.5	≥37.5
Cases, n (%)	83 (21.56)	153 (39.74)	96 (24.94)	53 (13.77)

**Table IV:** Changes in myocardial enzyme levels at 12 and 24 h following admission to hospital.

	CK, U/l		CK-MB, U/l		TNT, ng/ml		LDH, U/l		GOT, U/l	
					
Group	12 h	24 h	12 h	24 h	12 h	24 h	12 h	24 h	12 h	24 h
A	836.24±50.91	2844.12±271.62	109.69±34.21	237.92±40.74	1.65±0.36	3.99±1.47	417.85±61.48	637.85±86.45	134.07±59.14	296.89±71.29
B	869.45±69.47	2859.67±389.28	118.63±49.36^a^	236.47±75.16	1.78±0.56	4.11±1.54	426.23±78.15	622.17±102.38	146.29±66.57	329.13±72.14^a^
C	892.51±96.34^b^,^c^	3076.35±402.33^b^,^c^	143.15±36.17^a^	261.32±64.57^a^	1.77±0.49	3.97±1.93	452.36±71.66	636.93±135.27	151.26±53.69	322.26±82.39^a^
D	928.24±103.63^b^,^c^	3012.30±395.24^b^,^c^	135.03±31.25^a^	278.33±86.17^a^,^c^,^d^	1.98±0.65^a^	4.32±1.65	498.31±81.92^a^	712.31±141.21^a^	169.17±83.27^a^	347.49±87.18^a^

a P<0.05 and

b P<0.01, vs. group A;

c P<0.05, vs. group B;

d P<0.05, vs. group C.

CK, creatine kinase; CK-MB, creatine kinase-MB; TNT, troponin T; LDH, lactate dehydrogenase; GOT, glutamic-oxaloacetic transaminase.

**Table V:** Comparison of predominant cardiac function indexes.

Comparison of cardiac function among the groups at 24 h and 1 week following admission to hospital							

		LVEDV, ml		LVESV, ml		LVEF, %	
				
Group	Cases, n	24 h	1 week	24 h	1 week	24 h	1 week
A	83	123.54±35.66	138.36±36.18	61.04±26.55	73.74±29.95	55.09±9.68	53.22±10.26
B	153	126.47±32.61	140.23±40.52	61.47±20.73	74.17±27.27	54.23±8.69	52.32±11.29
C	96	131.18±36.43	137.42±36.78	62.46±19.37	75.36±30.49	53.31±8.33	52.78±9.71
D	53	127.53±35.26	136.40±37.59	63.57±23.48	74.78±33.12	53.16±9.71	51.27±10.63

a P<0.05, vs. group A.

CK-MB, creatine kinase-MB; LVEDV, left ventricular end-diastolic volume; LVESV, left ventricular end-systolic volume; LVEF, left ventricular ejection fraction.
